# Retraction Note: Tert-butylhydroquinone lowers blood pressure in AngII-induced hypertension in mice via proteasome-PTEN-Akt-eNOS pathway

**DOI:** 10.1038/s41598-019-53022-7

**Published:** 2019-11-19

**Authors:** Bing-Can Xu, Hui-Bao Long, Ke-Qin Luo

**Affiliations:** Department of Emergency, Sun Yat-Sen Memorial Hospital, Sun Yat-Sen University, Guangzhou, China

Retraction of: *Scientific Reports* 10.1038/srep29589, published online 20 July 2016

The editors retract this article.

It was brought to the editors’ attention that multiple figures in this article overlap with a paper entitled “Resveratrol rescues hyperglycemia-induced endothelial dysfunction via activation of Akt” which was published in Acta Pharmacologica Sinica in December 2016^1^.

Specifically, four subpanels in Figure 1B in this article are identical to Figures 1A and 1B in Ref 1. One subpanel in Figure 1E in this article is identical to a subpanel in Figure 1D in Ref 1. Four subpanels in Figure 2A in this article are identical to Figure 2A in Ref 1. Two subpanels in Figure 3B in this article are identical to Figure 3A in Ref 1. Two subpanels in Figure 3C in this article are identical to Figure 3B in Ref 1. Figures 2C, 2D, 4A, 4B, and 4C are identical between both articles. Figures 6A, 6B, 6C, and 6D in this article are identical to Figures 5A, 5C, 5D, and 5F, respectively, in Ref 1. Finally, Figure 8A in this article is identical to Figure 6B in Ref 1.

The editors requested the raw data from the authors, but received no response. The editors then liaised with the institution to obtain current contact information for the authors and were able to contact two of the three authors, who both confirmed that they were not aware of this submission.

Since the underlying raw data is not available, we are unable to establish that this work was carried out as presented. Additionally, the submission to the journal without the knowledge of two of the authors was in breach of Nature Research editorial policies. Taking all of this into account, the editors decided to retract this article.

Ke-Qin Luo could not be reached. Hui-Bao Long and Bing-Can Xu agreed to the retraction.
